# State Spending on Home and Community-Based Services and Rates of Low-Care Residents in Nursing Homes

**DOI:** 10.1093/geroni/igaf122.3433

**Published:** 2025-12-31

**Authors:** Blythe Chen, Elizabeth White

**Affiliations:** Brown University, Providence, Rhode Island, United States; Brown University School of Public Health, Providence, Rhode Island, United States

## Abstract

States are increasingly investing in home & community-based services (HCBS) to support older adults “aging in place” and prevent unnecessary nursing home (NH) admission of individuals with low care needs. This observational study examined associations between state HCBS spending (i.e. the percent of state Medicaid long-term care dollars spent on HCBS) and “low care” nursing home use in 2021 using the AARP LTSS State Scorecard and LTCFocus. In linear regression models controlling for state and NH characteristics, we estimated the association of state tertile of HCBS spending with percent of low-care residents in U.S. NHs (n = 14,105). The final model included an interaction between average state NH cost and HCBS spending. Compared to NHs in lowest-HCBS spend states, NHs in the highest-spend HCBS tertile had 1.08 percentage points (95% CI: -2.15, -0.01) fewer low-care residents. There was a significant interaction between state median NH cost and HCBS spending. In low-cost states, adjusted mean of low-care residents was 11.0% (95% CI: 10.55, 11.46) in lowest HCBS tertile, and 9.92% (95% CI: 9.47, 10.04) in highest HCBS tertile. In high-cost states, adjusted mean of low-care residents was 7.47% (95% CI: 6.94-8.00) in lowest HCBS tertile, and 9.93% (95% CI: 9.01-10.84) in highest HCBS tertile. Higher state HCBS spending is associated with a lower prevalence of low-care NH residents, but this relationship is attenuated in higher-NH cost states. Further research is needed to examine specific HCBS waiver-covered services associated with reducing NH admissions for low-care residents.

